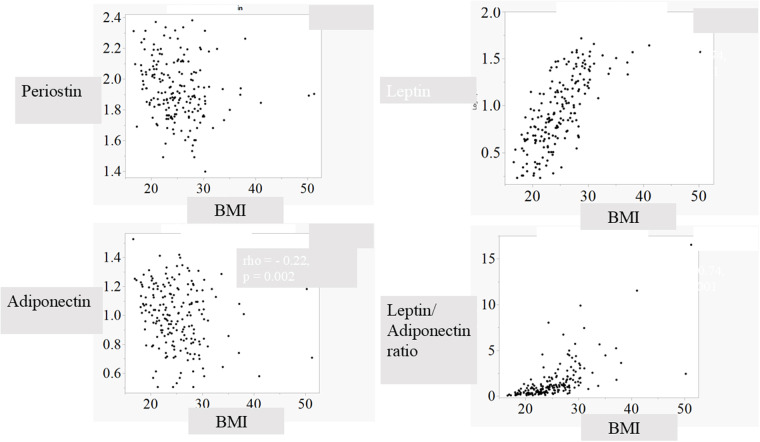# Determinants of airway morphology in asthma: Inflammatory and noninflammatory factors

**DOI:** 10.1016/j.jacig.2025.100555

**Published:** 2025-08-14

**Authors:** Kaoruko Shimizu, Naoya Tanabe, Hirokazu Kimura, Jun Miyata, Shotaro Chubachi, Yuji Nakamaru, Akira Oguma, Nobuyasu Wakazono, Kazufumi Okada, Houman Goudarzi, Ichizo Tsujino, Hironi Makira, Masaharu Nishimura, Satoshi Konno

**Affiliations:** a Department of Respiratory Medicine, Faculty of Medicine, Hokkaido University, Sapporo, Japan; b Department of Respiratory Medicine, Graduate School of Medicine, Kyoto University, Kyoto, Japan; c Division of Pulmonary Medicine, Department of Medicine, Keio University School of Medicine, Tokyo, Japan; d Department of Otolaryngology Head and Neck Surgery, School of Medicine, Hokkaido University, Sapporo, Japan; e Department of Respiratory Medicine, Oji General Hospital, Tomakomai, Hokkaido, Japan; f Data Science Center, Promotion Unit, Institute of Health Science Innovation for Medical Care, Hokkaido University Hospital, Sapporo, Japan; g Hokkaido Medical Research Institute for Respiratory Diseases, Sapporo, Japan

**Keywords:** Airway remodeling, asthma, body mass index, computed tomography, eosinophils, leptin, Lund-Mackay score, mucus plugs, obesity, type 2 inflammation

## Abstract

**Background:**

Patients with asthma may exhibit impaired airway tree morphology. The impact of difficult-to-treat traits on airway tree morphology remains unclear.

**Objective:**

We sought to identify determinants of total airway branch count (TAC) detectable via computed tomography and explore associated blood and sputum biomarkers in nonsmokers and smokers with asthma.

**Methods:**

Baseline computed tomography scans and pulmonary function tests (spirometry, diffusion capacity of carbon monoxide, and lung volume) were analyzed from the Hokkaido Severe Asthma Cohort (N = 190). TAC, segmental airway, visually evident mucus plugging and bronchiectasis, and parenchymal and extrapulmonary indices, such as the Lund-Mackay score, were evaluated. Relationships between TAC, difficult-to-treat traits, and blood/sputum biomarkers were analyzed using crude or multivariable regression models, adjusted for demographic factors.

**Results:**

Blood or sputum eosinophilia, mucus plugs, high body mass index (BMI), asthma duration, and higher Lund-Mackay score correlated with low TAC. Low TAC was linked to airflow obstruction and heterogeneous ventilation (low alveolar volume/total lung capacity). BMI was inversely associated with TAC, independent of age, sex, smoking status, sputum eosinophil ratio, and asthma duration. The presence of bronchiectasis correlated with an increase in TAC. Sputum IL-5, IL-6, RANTES, and circulating YKL-40 (chitinase-3-like-1 protein) and leptin also inversely correlated with TAC.

**Conclusions:**

BMI, asthma duration, sinusitis, and the presence of bronchiectasis are significant determinants of airway tree morphology in asthma, alongside inflammation and mucus plugs. Both inflammatory and noninflammatory biomarkers were associated with low TAC.

## Graphical abstract

**Figure:**
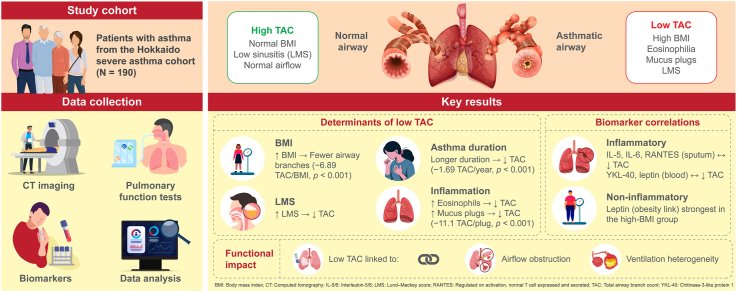

Asthma in some patients remains uncontrolled despite treatment,^1^^,^^2^ and these patients experience exacerbations, persistent symptoms, reduced quality of life (QoL), and impaired lung function, which are all treatable traits.^3^ Preserving lung function is a primary goal in the Global INitiative for Asthma guidelines^4^ and clinical remission strategies.^5^ Impaired lung function is linked to increased symptoms, poor QoL, and frequent exacerbations.^6^, ^7^, ^8^ Airway remodeling, inflammation, exudates, and mucus plugging contribute to airflow obstruction.^9^^,^^10^ Lung imaging, particularly computed tomography (CT), is vital for assessing proximal and small airway disease.^11^, ^12^, ^13^

Inspiratory scans comprehensively evaluate proximal airway tree morphology.^14^, ^15^ According to the Weibel model,^16^ the number of bronchi increases exponentially across generations. Given the heterogeneity of airway disease and the importance of smaller airways in airflow,^16^ global airway assessment may offer advantages over region-specific evaluations. Total airway branch count (TAC), a CT-based metric, assesses airway tree morphology.^11^^,^^15^ TAC mirrors pathologically suggested small airway disease in patients with mild chronic obstructive pulmonary disease (COPD). Regional airway alterations, such as parent airway wall thinning and luminal narrowing, reduce TAC.^11^ Furthermore, TAC is a promising predictor for assessing COPD risk or progression and, together with mucus plugs, may help predict functional decline, loss of independence, and mortality.^17^

Proximal airway disease has been studied on CT in patients with asthma. However, the pathophysiology of altered airway tree morphology requires further investigation.^18^ Reduced blood or sputum eosinophil count is associated with the degree of TAC reversal.^19^ Thus, type 2 airway inflammation may contribute to luminal narrowing and wall thickening of segmental airways, a measure of proximal airway disease, and alterations in distal airways.^19^ In addition, mucus plugs contribute to reduced TAC and airflow obstruction in COPD and asthma.^20^ IL-5 or IL-4/13 sputum levels correlate positively with higher mucus scores on CT.^21^ Collectively, the association between airway inflammation, mucus plug formation, and global airway structure, as assessed by CT, may exist in asthma.

Another distinct feature of airway tree morphology in asthma is severity dependency.^22^ Autopsy studies show that patients with severe asthma exhibit less airway complexity than healthy controls,^23^ which is supported by CT-based studies revealing decreased TAC in severe asthma.^22^ Factors that worsen asthma control (obesity, chronic rhinosinusitis, smoking, and prolonged asthma duration)^24^ may also be linked to impaired airway tree structure.^23^

Thus, we hypothesized that factors linked to difficult-to-treat asthma, such as obesity, prolonged asthma duration, and chronic rhinosinusitis, contribute to impaired airway tree morphology via inflammatory or noninflammatory pathways, leading to airway narrowing or obstruction, alongside eosinophilic inflammation or mucus plugging in patients with asthma. To test this hypothesis, we investigated the determinants of decreasing TAC and examined associated blood or sputum biomarkers in a cohort of smokers and nonsmokers with asthma.

## Methods

### Participants

This study was approved by the Ethics Committee of Hokkaido University Hospital (approval no. 009-0205) and was registered with the University Hospital Medical Information Network Clinical Trials Registry (UMIN-CTR) system (ID no. 000003254). Written informed consent was obtained from all participants before their inclusion in the study. All procedures involving human participants were conducted in accordance with the Declaration of Helsinki and institutional guidelines.

Patients with severe asthma who were a part of the Hokkaido-based Investigative Cohort Analysis for Refractory Asthma study^25^ were enrolled between February 2010 and September 2012 at Hokkaido University Hospital and 29 affiliated hospitals and clinics. Respiratory physicians confirmed asthma diagnoses. Patients with mild to moderate asthma were registered between March 2011 and September 2012. All participants were stable for at least 6 months without oral corticosteroid bursts. Severe asthma diagnoses met the 2000 American Thoracic Society (ATS) criteria for refractory asthma. Inhaler doses were adjusted on the basis of their availability in Japan. For a diagnosis according to the definition of severe asthma, 1 or both major criteria and at least 2 minor criteria should be met.

#### Major criteria

To achieve asthma control, there should be

1. Continuous or near-continuous oral corticosteroids (>50% of the year);
2. High-dose inhaled corticosteroids (ICSs), adjusted for availability in Japan: fluticasone propionate (≥800 μg), budesonide (≥1200 μg), beclometasone dipropionate (≥600 μg), ciclesonide (≥600 μg), mometasone furoate (≥600 μg), fluticasone propionate/salmeterol (≥1000 μg), and budesonide/formoterol (≥960 μg).

#### Minor criteria

The minor criteria included the following:

1. Daily treatment with a controller medication in addition to ICSs (eg, long-acting β-agonist, theophylline, or leukotriene antagonist);
2. Daily or near-daily asthma symptoms requiring short-acting β-agonist use;
3. Persistent airway obstruction (FEV_1_, <80% predicted; diurnal peak expiratory flow variability, >20%);
4. One or more urgent care visits for asthma annually;
5. Three or more oral steroid “bursts” per year;
6. Rapid deterioration with less than 25% reduction in inhaled or oral corticosteroid dose;
7. A near-fatal asthma event in the past.

ICS doses, oral corticosteroid use, and hospitalization in the previous year were assessed by reviewing prescriptions alongside clinical trials. Coordinators also interviewed participants regarding their tobacco pack years at entry.

Participants were categorized into those with severe and nonsevere asthma.

### Asthma Quality of Life Questionnaire (S) assessment and hospitalization during the year before the entry

Asthma-related QoL was assessed using the Japanese version of the Asthma Quality of Life Questionnaire (AQLQ (S))^26^ during the initial visit, with permission from Professor Elizabeth F. Juniper (McMaster University, Hamilton, Ontario, Canada).^26^ The AQLQ (S) comprises 4 categories: activity limitations (11 items), symptoms (12 items), emotional functioning (5 items), and exposure to environmental stimuli (4 items), for a total of 32 items, the average of which provides the AQLQ (S) score.^27^ Each item was rated on a scale from 1 (most impaired) to 7 (least impaired). Patients self-assessed their asthma-related QoL over the previous 2 weeks. The history of asthma-related hospitalization in the previous year was also assessed during the initial visit.

### Atopy

Atopy was defined as a positive specific IgE (>1.01 lumicount) for at least 1 common inhaled allergen, determined via a multiple-antigen simultaneous test.^25^

### Pulmonary function tests

Spirometry and diffusion capacity of carbon monoxide in the lungs (DLco) were evaluated using Chestac (Chest MI, Inc, Tokyo, Japan). Maintenance and calibration were followed according to the Japanese Respiratory Society guidelines.^28^ The best FEV_1_ and forced vital capacity (FVC) recorded during spirometry were documented as per the Japanese Respiratory Society guidelines. DLco was measured immediately after prebronchodilator spirometry using the single-breath method. The alveolar volume (*V*_A_)/total lung capacity (TLC) ratio served as an index for ventilation heterogeneity, with low *V*_A_/TLC indicating severe ventilation heterogeneity. DLco was corrected for hemoglobin concentration according to the European Respiratory Society/ATS guidelines^29^ using the Burrows prediction equation.^29^

Lung volumes (TLC, functional residual capacity, and residual volume [RV]) were assessed via the multiple-breath helium closed-circuit method. These volumes were expressed as percentages of the predicted values on the basis of the Nishida prediction equations.^30^

### Fractional exhaled nitric oxide

Fractional exhaled nitric oxide concentrations were measured using the NIOX MINO monitor (Circassia, Solna, Sweden), as per the ATS guidelines.^25^

### Quantitative CT

Participants who underwent full inspiratory CT scans using a standardized protocol with a 64-detector array (Aquilion Multi, TSX-101A/6A; Toshiba Medical Systems, Tochigi, Japan) at Hokkaido University Hospital were analyzed.^31^

### Intrapulmonary indices of airway and parenchymal disease

#### Total airway branch count

The airway tree was segmented and skeletonized, defining its centerline. Branching points were identified by the generation number of each branch, starting from the right and left main bronchi (generation 1) and extending to peripheral airway branches. This was performed using the Python package “Skan” and a custom script, with manual modifications as required. TAC was calculated by counting all branches of the skeletonized tree.^32^

#### Quantitative assessment of airway dimension and emphysematous regions

SYNAPSE VINCENT (Fuji Film, Tokyo, Japan) was used to quantitatively assess airway dimensions and emphysematous regions.^31^ The ratio of wall area to airway dimension (%WA) served as an index of airway remodeling. %WA values for the right apicalis (B1) and ventrobasalis (B8) at segmental or subsegmental airways were averaged. The ratio of low attenuation volume with a threshold of −950 HU to the total lung volume (%LAV) was used as an index of parenchymal disease.

#### Visual scoring of mucus plugs

Mucus plugs were visually assessed in all segments of the right lung, except for B7 (a total of 9 segments), without electrocardiogram synchronization,^33^ using the scoring system by Dunican et al.^21^ The mucus plug score reflects the number of segments with mucus plugs. A mucus plug is identified when an airway is completely obstructed in an area larger than 20 mm from the costal and diaphragmatic pleura.

The presence of bronchiectasis was visually determined according to the definition by Dunican et al.^21^ Concordant with the assessment of mucus plugs, the presence of bronchiectasis was assessed only in the right lungs because of motion artifacts in the left lungs caused by cardiac motion. The presence of bronchiectasis was confirmed when the ratio of bronchi to the accompanying artery was more than 1.5.

### Extrapulmonary indices of visceral and subcutaneous fat and sinus findings

#### Lund-Mackay score

An experienced otolaryngologist (Y.N.) evaluated the sinus CT images using the Lund-Mackay score (LMS).^18^^,^^34^^,^^35^ The 5 sinus complexes—maxillary, anterior/posterior ethmoidal, sphenoidal, and frontal—were scored on a scale of 0 to 2 (0, completely aerated; 1, partially opacified; and 2, completely opacified). The osteomeatal complex was rated as 0 (unobstructed) or 2 (obstructed). Sinus CT images received an overall score ranging from 0 to 24.

#### Visceral and subcutaneous fat

Along with chest and sinus CT images, an abdominal CT scan was performed at the level of the third lumbar vertebra with the patient in the supine position. The Fat Scan software program Ziostation 2 (Ziosoft, Inc, Tokyo, Japan)^5^ was used to quantify visceral and subcutaneous fat areas from the CT images.^36^

### Blood biomarkers, cell counts, and cytokine/chemokine levels in sputum supernatant

The serum levels of 4 blood biomarkers previously associated with asthma, sinusitis, and obesity—periostin, human chitinase-3-like-1 protein (YKL-40), leptin, and adiponectin^37^, ^38^, ^39^—were selected and measured by ELISA.

For sputum evaluation, a 2-fold volume of 0.1% dithiothreitol/PBS was added to a 50-mL polystyrene tube containing induced sputum. The tubes were shaken in a water bath for 30 minutes at 37°C. After adding 2-fold volume of PBS, the shaking continued for another 10 minutes. Following centrifugation, the supernatant was collected. At least 250 nonsquamous cells were assessed per smear.

For cytokine assessment, sputum supernatant samples were immediately frozen after collection and stored until analysis. Concentrations of 21 molecules were measured in 30 samples using the Luminex multianalyte technology (Luminex Corp, Austin, Tex) and specific ELISAs (R&D Systems, Minneapolis, Minn). Analytes with more than 50% undetectable values were excluded, including IL-13. Fourteen molecules, including eotaxin, IL-5, IL-6, IL-12, and RANTES, were measured in all patients. All samples were measured in duplicate. For undetected values, half the sensitivity limit was assigned.^25^

All blood and sputum biomarkers were stored in a freezer at −80°C.

### Statistical analysis

To examine factors associated with low TAC, the following variables were compared: anthropometric indices, smoking status, asthma severity and duration, fractional exhaled nitric oxide, IgE, use of inhaled and oral corticosteroids, eosinophil and neutrophil counts in blood, sputum eosinophil and neutrophil ratios, AQLQ (S) scores, pulmonary function test values, and CT-related parameters. Variables were analyzed across groups stratified by TAC (low, <25th percentile; moderate, 25th < TAC < 75th percentile; and high, >75th percentile), using the Student *t* test, Wilcoxon test, or chi-square tests. Associations among TAC, pulmonary function indices, and blood/sputum biomarkers were assessed using the Spearman correlation coefficient. Multiple regression analysis evaluated associations of TAC with age, sex, pack years of tobacco use, and asthma severity. Relationships between body mass index (BMI), blood and sputum biomarkers, and mucus plugs were compared across BMI categories (high, >25; moderate, 22.5-25; and low, <22.5), reflecting the generally lower BMI in the Japanese population. All statistical analyses were performed using the JMP software (SAS Institute, Cary, NC).

## Results

**Fig 1:**
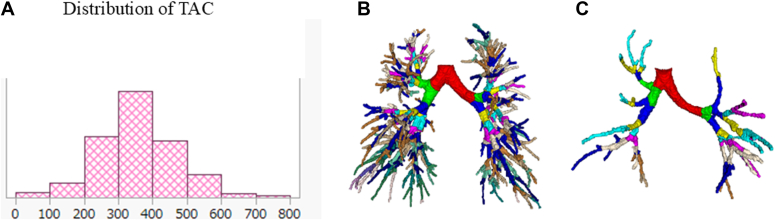
Distribution of TAC and representative CT images of patients with asthma. **A,** TAC follows a normal distribution in both smokers and nonsmokers with asthma. **B** and **C,** Representative CT images for a female patient with high TAC (TAC, 641; FEV_1_, 114.8%; FEV_1_/FVC, 90.0%) (Fig 1, *B*) and another female patient with low TAC (TAC, 89; FEV_1_, 70.6%; FEV_1_/FVC, 48.8%) (Fig 1, *C*).

Fig 1, *A*, illustrates the distribution of TAC, which follows a normal distribution in patients with asthma. Representative CT images were shown for a female patient with high TAC (TAC, 641; FEV_1_, 114.8%; FEV_1_/FVC, 90.0%) (Fig 1, *B*) and another female patient with low TAC (TAC, 89; FEV_1_, 70.6%; FEV_1_/FVC, 48.8%) (Fig 1, *C*).

**Supplementary Fig E1:**
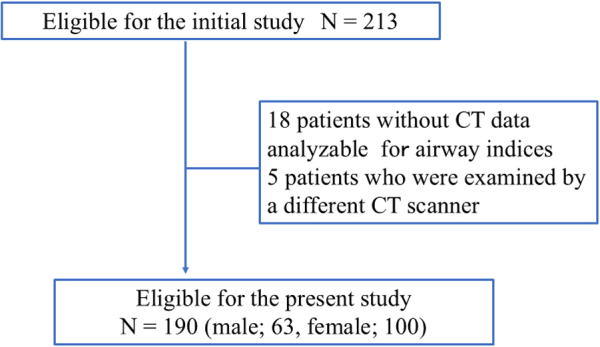


Fig E1 (in the Online Repository available at www.jaci-global.org) shows the participant flowchart. Of the 213 patients, 18 were excluded because of nonanalyzable CT data, and 5 were excluded because a different CT machine was used.

### Comparisons between TAC-defined groups

**Fig 2:**
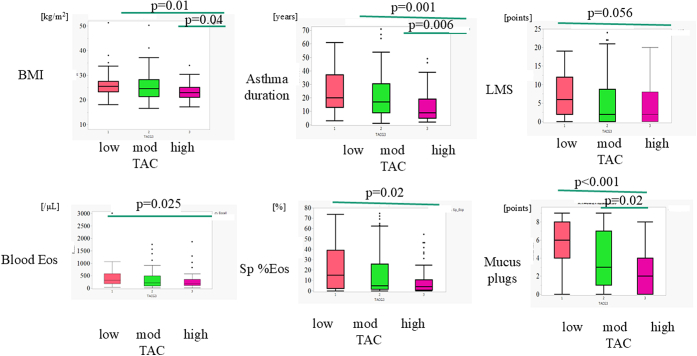
Possible determinants for airway tree morphology: BMI, asthma duration, LMS, blood/sputum eosinophilia, and mucus plugs among TAC-based groups. TAC-based group comparisons include BMI, asthma duration, LMS, eosinophilia in blood or sputum, and mucus plug scores. *Sp%Eos*, Percentage of eosinophils in sputum.

Tables I and II present comparisons of demographic characteristics, asthma-related variables, and physiological and radiological indices among TAC-defined groups (low-TAC group [N = 47]; moderate-TAC group [N = 96]; and high-TAC group [N = 47]). Participants in the low-TAC group were older and had a higher BMI, longer asthma duration, higher mucus plug score, and lower %FEV_1_, FEV_1_/FVC, and *V*_A_/TLC compared with those with high TAC. They also had a higher percentage transfer coefficient of carbon monoxide and RV/TLC compared with the high-TAC group. Regarding CT-related indices, %WA of the third and fourth airway generations, the LMS, and visceral fat were more pronounced in the low-TAC group than in the high-TAC group. However, %LAV did not differ significantly between groups. Blood eosinophil count and sputum eosinophil ratio were higher in the low-TAC group (Fig 2). The moderate-TAC group exhibited higher BMI, prolonged asthma duration, and higher mucus plug score than the high-TAC group.

**Table I:** Characteristics of the participants in TAC-defined groups

Characteristics	Low TAC (N = 47)	Moderate TAC (N = 97)	High TAC (N = 47)
Sex: female, n (ratio, %)	30 (63.8)	62 (64.6)	23 (48.9)
Age (y)	68 (55, 72)∗	64 (55, 70.8)	57 (46, 71)
BMI (kg/m^2^)	25.5 (23.3, 27.6)∗	24.6 (21.4, 28.3)∗	22.9 (21.0, 25.1)
Severity, n (%)	35 (74.5)	64 (66.7)	28 (59.6)
PY > 10, n (%)	16 (34.0)	37 (38.5)	17 (36.2)
Duration (y)	20.5 (13, 36.8)∗	17 (9, 30.5)∗	9 (5, 19)
Atopy, n (%)	18 (38.3)	32 (33.3)	13 (27.7)
IgE (IU/mL)	159.9 (65.2, 536.4)	189.3 (57.9, 445.3)	134.2 (55.6, 385.7)
Feno (ppb)	41 (22.3, 62.5)	24 (14, 40.8)	28 (14, 49)
Eos (/μL)	318 (173, 579.8)∗	208 (86, 494)	179 (112, 351)
Neutro (/μL)	4270.5 (3239.1, 5538)∗	4051.7 (2953.0, 5857.4)∗	3408 (2672.6, 5564.2)
Sp%Eos	15.2 (2.4, 38.4)∗	4.8 (1.5, 26.0)	4.2 (0.8, 10.7)
Sp%Neutro	56 (34.3, 74.9)	56 (34.6, 73.8)	57.2 (39.2, 77.8)
ICS dose (μg)	1500 (800, 1600)	1500 (750, 1600)	1500 (750, 1500)
OCS, n (ratio, %)	12 (25)	23 (24)	11 (23)
AQLQ (points)	5.6 (4.9, 6.3)	5.8 (5.1, 6.4)	6.0 (5.0, 6.5)
Hospitalization, n (%)∗	33 (68.7)	43 (44.8)	18 (38.3)

When factors show non-normalized distribution, the lowest and highest quatiles are suggested. *Eos*, Blood eosinophil count; *Neutro*, blood neutrophil count; *OCS*, oral corticosteroid; *PY*, pack year of tobacco smoked; *Sp%Eos*, percentage of eosinophils in sputum; *Sp%Neutro*, percentage of neutrophils in sputum.

∗ A statistical significance compared with the high-TAC group.

**Table II:** Pulmonary function and CT-based indices in TAC-defined groups

Indices	Low TAC	Moderate TAC	High TAC
FEV_1_ (%)	89.3 (77.6, 101.8)∗	103.8 (92.5, 122.2)∗	121.9 (110.1, 141.0)
FEV_1_/FVC (%)	56.1 (48.6, 63.3)∗	69.3 (60.9, 77.1)∗	77.6 (71.6, 82.4)
DLco (%)	100.1 (87.5, 121.6)	100.8 (87.8, 117.1)	101.8 (89.3, 117.7)
Kco (%)	114.4 (96.6, 132.9)∗	109.0 (94.6, 119.4)	104.6 (90.0, 113.4)
RV/TLC (%)	39.2 (34.9, 42.9)∗	37.2 (32.2, 41.2)∗	31.8 (27.6, 37.4)
TLC (%)	109.8 (99.9, 121.8)	110.6 (102.6, 118.0)	113.0 (101.2, 123.6)
*V*_A_/TLC (%)	71.0 (0.67.7, 74.4)∗	73.1 (70.4, 76.1)	74.9 (71.9, 77.4)
Annual changes in FEV_1_ (mL)	−32.2 (−44.6, −15.2)	−33.1 (−44.3, −23.0)	−32.2 (−53.4, −19.4)
3rd%WA	57.7 (51.7, 64.2)∗	56.4 (50.0, 62.1)∗	50.2 (45.0, 55.5)
4th%WA	67.8 (64.3, 70.4)∗	64.8 (62, 67.7)∗	61 (57.1, 63.5)
%LAV	0.41 (0.1, 1.2)	0.4 (0.1, 2.2)	0.3 (0.1, 0.7)
Mucus plug score	6 (4, 8)∗ (N = 44)	3 (1, 7)∗ (N = 80)	2 (0, 4) (N = 39)
LMS (points)	6.5 (2, 11.8)∗	2 (0, 8.8)	2 (0, 8)
Visceral fat (cm^2^)	159.9 (117.6, 226.8)∗	151.4 (96.7, 208.3)	118.0 (82.9, 161.1)
Subcutaneous fat (cm^2^)	197.1 (133.0, 297.0)	210.2 (134.3, 188.2)∗	152.1 (106.3, 224.5)

A quartile range is suggested with a median value for each index. *Kco*, Transfer coefficient of carbon monoxide.

∗ A statistical significance compared with the high-TAC group.

**Supplementary Fig E2:**
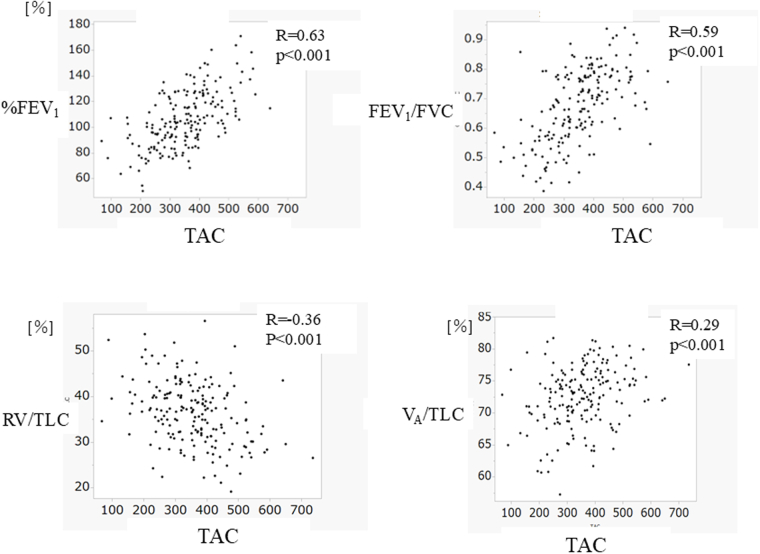


TAC was positively correlated with %FEV_1_, FEV_1_/FVC, and *V*_A_/TLC and negatively correlated with RV/TLC (see Fig E2 in this article’s Online Repository at www.jaci-global.org). Multivariable analysis revealed a significant negative association between BMI (Estimate, −6.89; 95% CI, −10.0 to −3.74) and TAC after adjusting for age, sex, pack years of tobacco use, severity of asthma, sputum eosinophil ratio, and asthma duration. This significant correlation persisted when the mucus plug score replaced the sputum eosinophil ratio in the model (Estimate, −7.67; 95% CI, −10.8 to −4.54) (Table III).

**Table III:** Multivariable analysis for determining the factors associated with TAC

Factors	Model 1		Model 2	
	Estimate (95% CI)	*P* value	Estimate (95% CI)	*P* value
BMI	−6.89 (−10.0 to −3.74)	<.001	−7.67 (−10.8 to −4.54)	<.001
Asthma duration	−1.69 (−2.69 to −0.70)	<.001	−1.67 (−2.65 to −0.68)	.001
Sp%Eos	−1.30 (−2.10 to −0.50)	.002		
Mucus plug score			−11.1 (−16.1 to −6.18)	<.001

*Note*. Both models are adjusted for age, sex, pack year of tobacco use, and severity of asthma (severe or nonsevere).

*Sp%Eos*, Percentage of eosinophils in sputum.

Bronchiectasis was present in 99 patients (61.1%), whereas 63 patients (38.9%) showed no bronchiectasis. TAC was significantly higher in patients with bronchiectasis (mean ± SD, 378.6 ± 109.8) than in those without bronchiectasis (mean ± SD, 299.7 ± 99.7) (*P* < .001). In a multivariate model, the presence of bronchiectasis was positively associated with an increase in TAC after adjusting for age, sex, smoking status (pack year), severity of asthma, asthma duration, sputum eosinophil ratio, and BMI. There was no significant difference in LMS or severity of asthma between patients with or without bronchiectasis. Longitudinal data are available in patients with severe asthma; the frequency of exacerbations in 3-year follow-ups was not significantly different between patients with (N = 35) and without bronchiectasis (N = 58). However information on childhood infections is not available for our asthma cohort. Moreover, there were 22 (11.6%) patients with diabetes who were undergoing treatment. No significant difference was found in the severity of asthma between patients with and without diabetes.

### Correlations of blood/sputum biomarkers with TAC

**Fig 3:**
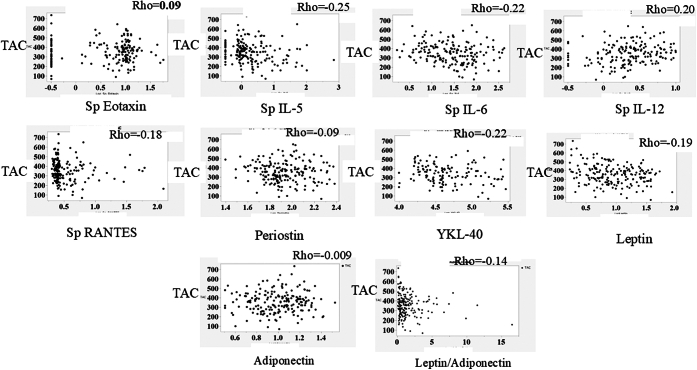
Relationships of TAC with sputum and circulating biomarkers in patients with asthma. TAC was inversely associated with sputum levels of IL-5, IL-6, IL-12, and RANTES as well as circulating levels of YKL-40 and leptin. TAC showed weak or no association with eotaxin, periostin, adiponectin, or the leptin/adiponectin ratio. Biomarker values were transformed using a logarithmic function.

To investigate the pathophysiology of TAC reduction, correlations between TAC and sputum eosinophil ratio, mucus plug score, BMI, and blood/sputum biomarker levels were analyzed. Sputum levels of IL-5 (ρ = −0.25; *P* < .001), IL-6 (ρ = −0.22; *P* < .01), IL-12 (ρ = 0.20; *P* = .009), RANTES (ρ = −0.18; *P* = .01), as well as circulating YKL-40 (ρ = −0.22; *P* < .01) and leptin (ρ = −0.19; *P* < .01) were correlated with TAC. The relationships of TAC with sputum levels of eotaxin, circulating periostin, adiponectin, and leptin/adiponectin (ρ = 0.09, *P* = .24; ρ = −0.09, *P* = .23; ρ = −0.009, *P* = 0.90; and ρ = −0.14, *P* = .06, respectively) were also analyzed (Fig 3).

### Comparisons of inflammation and mucus plugs among BMI-based groups

**Supplementary Fig E3:**
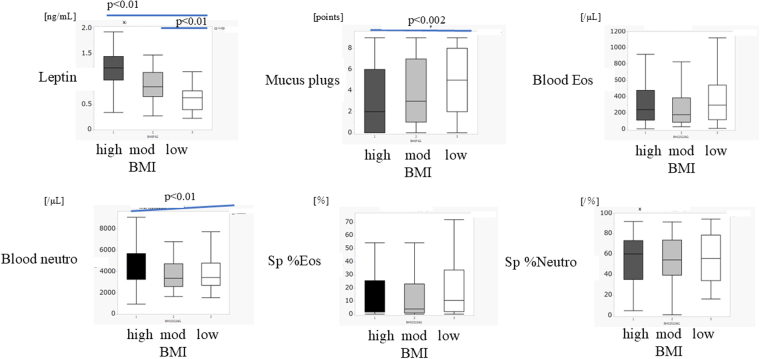


The level of leptin was the highest in patients with high BMI (N = 87), followed by those with moderate BMI (N = 44) and low BMI (N = 59). Mucus plugs were fewer in the high-BMI group (N = 73) than in the low-BMI group (N = 50). In addition, blood neutrophil count was higher in the high-BMI group compared with the low-BMI group, whereas blood or sputum eosinophilia and sputum neutrophilia showed no significant differences among the 3 groups. Notably, leptin levels varied significantly across the groups (see Fig E3 in this article’s Online Repository at www.jaci-global.org). The relationships between BMI and sputum levels of IL-5 (ρ = −0.007; *P* = .93), IL-6 (ρ = 0.07; *P* = .33), IL-12 (ρ = −0.002; *P* = .98), RANTES (ρ = −0.06; *P* = .41), eotaxin (ρ = −0.09; *P* = .22), circulating YKL-40 (ρ = 0.14; *P* = .13), circulating periostin (ρ = −0.24; *P* < .001), leptin (ρ = 0.74; *P* < .001), adiponectin (ρ = −0.22; *P* = .002), and leptin/adiponectin (ρ = 0.74; *P* < .001) are shown in Fig E4 (in the Online Repository available at www.jaci-global.org).

## Discussion

TAC negatively correlated with BMI, asthma duration, sinus and mucus plugs on CT, and sputum eosinophil ratio. Multivariable analysis showed that BMI was independently associated with reduced TAC, sputum eosinophil ratio, mucus plugging, and asthma duration. Sputum IL-5 levels were significantly correlated with the mucus plug scores. Sputum IL-5, IL-6, RANTES, and circulating YKL-40 and leptin inversely correlated with TAC, suggesting inflammatory and noninflammatory regulation.

BMI was a determinant of airway tree morphology, complementing other difficult-to-treat traits in asthma. Obesity has a significant impact on asthma complexity,^40^ with obese individuals experiencing more severe symptoms, poorer treatment response, and impaired QoL. Moreover, obesity reduces functional RV and diminishes airway conductance, indicating smaller airway caliber irrespective of asthma status.^41^ BMI, rather than waist circumference, has been reported to be associated with airway narrowing during methacholine-induced bronchoconstriction,^42^ potentially explaining mechanical susceptibility to stimuli. Excessive airway collapse has been linked to high BMI regardless of asthma status. Pathological studies have also identified adiposity within airway structures in obesity-linked asthma.^43^ Our previous study showed that high BMI was associated with poorer asthma outcomes mediated by reduced %FEV_1_, gastroesophageal reflux disease, and depression, highlighting the clinical importance of addressing physical factors.^36^ Bronchiectasis plays a vital role in airway morphology in patients with asthma. In this study, comorbid bronchiectasis was associated with an increase in TAC, whereas sinusitis findings on CT (LMS), severity of asthma, and exacerbations did not differ between patients with and without bronchiectasis. The impact of bronchiectasis on airway morphology was preserved after adjustment for age, sex, smoking status, severity of asthma, BMI, asthma duration, and sputum eosinophil percentage. Thus, combined assessment of quantitative total airway count and visual bronchiectasis enhances the interpretation of airway tree structures.

Low TAC correlated with low %FEV_1_, FEV_1_/FVC, RV/TLC, and *V*_A_/TLC. TAC is the number of airway branches detectable on CT, and higher TAC may suggest that more distal airway branches are large enough to be visible within CT resolution. Thus, TAC likely reflects the physiology of more distal airways, including small airway disease, as indicated by its association with RV/TLC. Notably, low TAC was correlated with low *V*_A_/TLC, suggesting ventilation heterogeneity, highlighting the utility of TAC in asthma management. Specifically, TAC reversal might reflect improvements in heterogeneous ventilation.

We found that TAC was negatively associated with sputum levels of IL-5, IL-6, IL-12, RANTES, circulating YKL-40, and leptin. Sputum IL-5 and RANTES (type 2 inflammation markers), along with airway eosinophilia, may contribute to airway disease, as reported previously. Another study reported that circulating YKL-40 was associated with airway fractal dimension and annual decline in FEV_1_ in the 5-year follow-up of patients with severe asthma.^38^ Thus, the correlation between TAC and YKL-40 supports the role of YKL-40 in airway tree morphology. Sputum IL-13 levels were undetectable in this study. However, higher levels of sputum IL-13 have been reported in patients with asthma who demonstrated more mucus plugs on chest CT than those with fewer mucus plugs.^21^ Thus, it is possible that patients with low TAC show increased sputum IL-13 levels. High sputum IL-6 and IL-12 levels might reflect non–type 2 inflammation. Notably, increased circulating leptin^44^ was related to low TAC. Obese patients tend to exhibit higher leptin levels and blood neutrophilia but not eosinophilia in blood or sputum, nor sputum neutrophilia. These findings suggest that BMI-related airway alterations involve regulation across inflammatory and noninflammatory pathways. A recent study demonstrated an association between low muscle density on CT, central airway wall thickening, and reduced airway tree complexity,^45^ further supporting the role of BMI in asthma. Given the small number of patients with diabetes in this study, further research is needed to elucidate metabolic pathways affecting airway structures. Caution is needed when interpreting the low mucus plug score in the high-BMI group. This finding does not imply a direct link between high BMI and decreased mucus plugs but may be explained by lung compression leading to narrowed airways and reduced CT-assessed mucus plug scores.

The link between upper airway disease, assessed by LMS, and lower TAC was confirmed in this study. Previous studies have shown that airway tree morphology, assessed through fractal dimension, correlates with LMS in asthma.^46^ Another study demonstrated that patients with high LMS exhibited low FEV_1_ and prominent type 2 inflammation.^25^ Furthermore, LMS affected asthma-related QoL, complementing the ratio of abdominal visceral fat to erector spinae muscle, airway tree morphology, and parenchymal disease.^46^ Together, these findings support the concept of “one airway, one disease” and highlight the contribution of upper airway disease to intrapulmonary disease and QoL in asthma. Thus, optimal control of type 2 inflammation and comprehensive airway management may be beneficial. Long-term observational and interventional studies are needed to clarify the pathophysiology of the upper and lower airways.

Prolonged asthma duration^47^ may reduce TAC, suggesting the accumulated burden of asthma on airways. Patients in this cohort adhered to prescribed medications at baseline. However, airway disease may progress during periods of suboptimal treatment in long-term asthma. Alongside anti-inflammatory treatments, bronchodilators mitigate bronchoconstriction and mechanical stress, reducing airway deformation. Obesity exacerbates reduced lung volumes, and repeated small airway closure during tidal breathing may cause trauma, leading to inflammation and remodeling.^46^

This study had limitations. First, the sample size was relatively small; however, the heterogeneity in smoking status and asthma severity enhanced generalizability. Second, the cross-sectional design limited longitudinal insights. Future prospective studies assessing airway tree morphology are needed to elucidate the etiopathogenesis of asthma.

Airway tree morphology may be impaired by type 2 inflammation, high BMI, prolonged asthma duration, and upper airway disease, with regulation dependent on inflammatory and noninflammatory pathways. These findings emphasize the importance of comprehensive asthma management.

**Key messages**

• **High BMI is independently associated with reduced airway branch counts, suggesting that obesity-related pathways contribute to airway morphology in asthma beyond inflammation.**
• **Global airway assessments (eg, TAC) provide valuable mechanistic insights into asthma pathophysiology and may help guide personalized therapeutic strategies.**
• **Prolonged asthma duration, sinusitis, and the presence of bronchiectasis correlate with impaired airway morphology, underscoring the need for comprehensive asthma management addressing comorbidities.**

## Disclosure statement

This work was supported by the 10.13039/501100001700Ministry of Education, Culture, Sports, Science and Technology of Japan (grant nos. 24249049 and 26461151); the Japan Allergy Foundation; AstraZeneca K.K; and 10.13039/100019271Kyorin Pharmaceutical Co, Ltd.

Disclosure of potential conflict of interest: K. Shimizu was supported by grants from Daiwa Health Development, Inc, outside the submitted work. N. Tanabe was supported by grants from FUJIFILM Co, Ltd, and Daiichi Sankyo Co, Ltd; and received honoraria from AstraZeneca, outside the submitted work. J. Miyata was supported by grants from GlaxoSmithKline; and received payment for lectures from GlaxoSmithKline, AstraZeneca, and Sanofi, outside the submitted work. Y. Nakamaru received payment for lectures from Sanofi Co, Ltd. I. Tsujino was supported by grants from Mochida Pharmaceuticals K.K., Nippon Shinyaku Co, Ltd, Nippon Boehringer Ingelheim Co, Ltd, Medical System Network Co, Ltd, Kaneka Corp, and Takeyama Co, Ltd; and received honoraria from Nippon Shinyaku Co, Ltd, and Janssen Pharmaceutical K.K., outside the submitted work. S. Konno was supported by grants from Mochida Pharmaceuticals K.K., Nippon Shinyaku Co, Ltd, Nippon Boehringer Ingelheim Co, Ltd, Medical System Network Co, Ltd, Kaneka Corp, Takeyama Co, Ltd, and Novartis; and received honoraria from AstraZeneca and KYORIN Pharmaceutical, outside the submitted work. The rest of the authors declare that they have no relevant conflicts of interest.

## Figures and Tables

**Supplementary Fig E4:**